# DG-Affinity: predicting antigen–antibody affinity with language models from sequences

**DOI:** 10.1186/s12859-023-05562-z

**Published:** 2023-11-13

**Authors:** Ye Yuan, Qushuo Chen, Jun Mao, Guipeng Li, Xiaoyong Pan

**Affiliations:** 1 Institute of Image Processing and Pattern Recognition, Shanghai Jiao Tong University, and Key Laboratory of System Control and Information Processing, Ministry of Education of China, Shanghai, 200240 China; 2 DigitalGene, Ltd, Shanghai, 200240 China

**Keywords:** Affinity, Deep learning, Sequence embedding, Antibody–antigen interaction

## Abstract

**Background:**

Antibody-mediated immune responses play a crucial role in the immune defense of human body. The evolution of bioengineering has led the progress of antibody-derived drugs, showing promising efficacy in cancer and autoimmune disease therapy. A critical step of this development process is obtaining the affinity between antibodies and their binding antigens.

**Results:**

In this study, we introduce a novel sequence-based antigen–antibody affinity prediction method, named DG-Affinity. DG-Affinity uses deep neural networks to efficiently and accurately predict the affinity between antibodies and antigens from sequences, without the need for structural information. The sequences of both the antigen and the antibody are first transformed into embedding vectors by two pre-trained language models, then these embeddings are concatenated into an ConvNeXt framework with a regression task. The results demonstrate the superiority of DG-Affinity over the existing structure-based prediction methods and the sequence-based tools, achieving a Pearson’s correlation of over 0.65 on an independent test dataset.

**Conclusions:**

Compared to the baseline methods, DG-Affinity achieves the best performance and can advance the development of antibody design. It is freely available as an easy-to-use web server at https://www.digitalgeneai.tech/solution/affinity.

**Supplementary Information:**

The online version contains supplementary material available at 10.1186/s12859-023-05562-z.

## Background

Antibody-mediated immune response is a central component of human immune system. Antibodies are a special protein that can specifically recognize invading antigens, such as viruses, by binding to epitopes on the antigens through the two ends of their Y-shaped structure, known as the complementarity-determining regions (CDRs) [1–3]. Due to the high diversity of CDRs, they show binding specificity toward specific antigens [4]. The biopharmaceutical industry has utilized this specificity to develop monoclonal antibodies (MAbs) as therapeutic drugs, which have high success rates and efficacy for diseases. In addition, they suffer to minimal side effects [5–8]. With the advancement of biotechnology techniques, such as antibody–drug conjugates (ADCs), even traditional "undruggable" targets of diseases can be targeted. Antibodies can be used to treat various cancers, as well as autoimmune diseases like rheumatoid arthritis, attracting huge research attention and development efforts [8–12]. Since the approval of the first monoclonal antibody, antibodies have become popular drugs, occupying more than half of the therapeutic market [13]. The latest application of monoclonal antibodies is the treatment of the 2019 coronavirus disease (COVID-19), since some patients may not be suitable for vaccination due to severe allergic reactions or inability to generate protective immune responses from the vaccine. Recently, monoclonal antibodies against SARS-CoV-2, such as bebtelovimab, tixagevimab and cilgavima, have been approved by the FDA for the treatment or pre-exposure prevention of COVID-19, demonstrating that monoclonal antibodies can be an effective complement to vaccines against COVID-19 [14–21].

Determining the affinity of antibody–antigen interactions is an important step in antibody development. Experimental methods for affinity determination include radioimmunoassay (RIA), enzyme-linked immunosorbent assay (ELISA), surface plasmon resonance (SPR), and bio-layer interferometry (BLI) [22–26]. However, some of these experimental methods are resource-intensive and time-consuming. Moreover, these experimental methods are not suitable for large-scale high-throughput antibody screening [27]. Fortunately, extensive immunological databases from experiments have been established, generating a wealth of experimental affinity data for antigen and antibody studies [28–33]. With the advancement of artificial intelligence technologies, especially, deep learning performs better than traditional machine learning methods on large datasets. For example, ConvNeXt outperforms the Swin-T model in multiple classification and recognition tasks. The model with ConvNeXt as the backbone has also achieved good results in fields such as medical imaging and traditional Chinese medicine. It has become possible to build predictive models based on these collected data and deep learning methods to predict antibody–antigen affinity [34–36] with high accuracy. For example, PIPR is a sequence-based method and employs a residual RCNN [37] to predict binding affinity using information from antigen–antibody pairs. It achieves good generalization performance on various tasks. The RCNN framework in PTPR adopts a bidirectional gated recursive unit module (GRU), however, GRU has the drawbacks of slow learning efficiency and convergence speed [38]. Another model is the CSM-AB model [39], it first requires docking of antibody and antigen structures or utilizes known complex structures, and then obtains geometric information of the contact interface to establish a predictive model using Extra Trees algorithm. Recently, the AREA-AFFINITY was developed to predict antibody–antigen binding affinity [40]. It built different models including linear model, neural network, random forest and mixed model. The mixed model yields the best performance than other compared methods. Similar to CSM-AB, the AREA-AFFINITY is also a structure-based model. However, the limitation lies in the requirement for antigen–antibody complex structure information, which is difficult to acquire.

In this study, we propose a sequence-based method DG-Affinity for predicting antibody–antigen binding affinity. It is trained on a larger and more comprehensive dataset than CSM-AB, and only utilizes sequence information to predict the affinity between antibodies and antigens. DG-Affinity combined two pre-trained embeddings (TAPE for antigen sequences and Ablang for antibody sequences) on an antibody–antigen interaction dataset, and used a ConvNeXt framework [41] to learn the relationship of antibody–antigen binding affinity. DG-Affinity outperforms other existing methods in an independent test dataset.

## Materials and methods

### Benchmark datasets

The benchmark antigen–antibody data comes from two primary sources. One is the sdAb-DB database (http://www.sdab-db.ca) [42], which is a freely available repository that collects antibody–antigen data. The other is the Round A data from the Baidu PaddlePaddle 2021 Global Antibody Affinity Prediction Competition. In this study, we combine the two datasets by removing the shared antibody–antigen interactions to construct the benchmark dataset. The benchmark dataset comprises 1,673 entries involving 448 distinct antibody–antigen complexes [43].

We divided the data into five equal parts. Four parts were used for training and one for validation, take turns to conduct five times of cross-validation. Then, we utilized an independent test set to evaluate the model’s generalizability. This independent test set came from (https://github.com/piercelab/antibody_benchmark) [33], which contains structural files for 42 antigen–antibody complexes with affinity values not overlapping with the training data. We selected structures where both the antibody and the antigen are single chains, totaling 26, and used the PDB module in Biopython to extract sequence information from the complex structures as the independent test set [44].

**Fig. 1:**
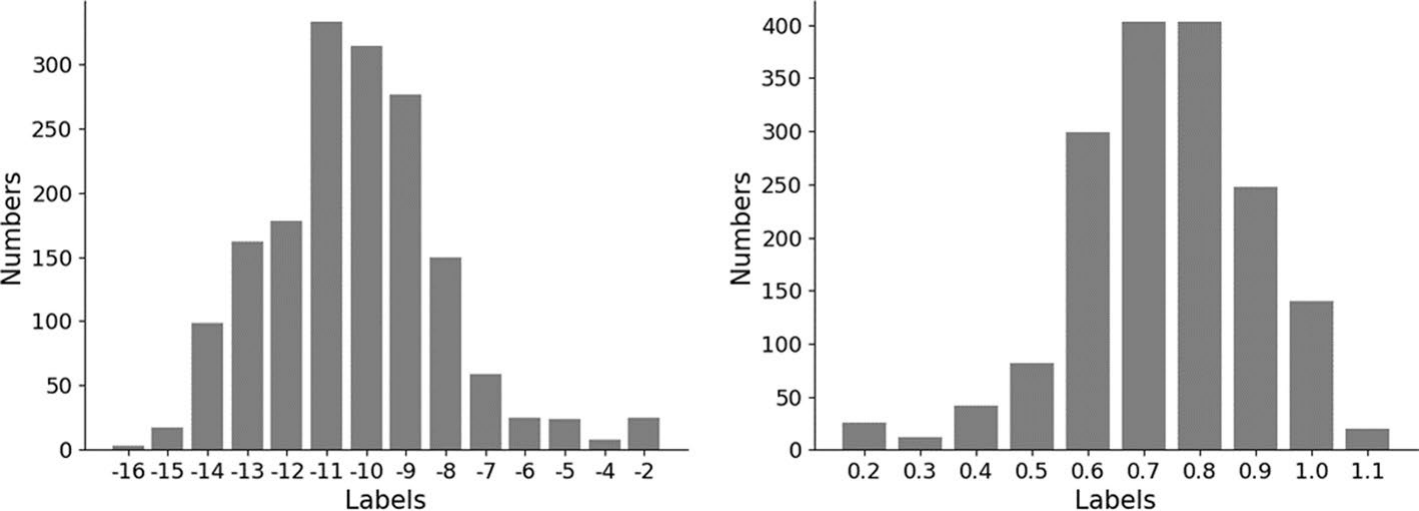
Distribution of affinity values before and after data preprocessing. **a** Before preprocessing and **b** after preprocessing. The x-axis represents the data label value and the y-axis represents the numbers of data within this value range

We processed the data by treating each antibody–antigen pair and its corresponding affinity label separately. As shown in Fig. 1a, the value range of the original dissociation constant (kd) value of the binding affinity is from − 2 to − 16, and was then transformed by taking the negative logarithm, as used in [45], and dividing by 10 for normalization as follows:

$$y_{label} = \frac{{ - (y_{kd} *1000)/591.282}}{{log_{e} \left( {10} \right)/10}}$$

where $y_{kd}$ is the original value and 591.82 is from the study [45].

As shown in Fig. 1b, it can be clearly seen that the original kd value was successfully converted to the range of 0–1, and only a small number of abnormal data values were mapped beyond 1.

### Sequence embedding of antibody and antigen

For the antigen sequence embeddings, we used TAPE’s pre-trained model to obtain [46], a protein language model, the embeddings. A protein language model is a type of language model designed for the protein sequences. It is trained on protein sequences and learns underlying biochemical properties, secondary and tertiary structures, and intrinsic functional patterns.

TAPE uses bi-directional encoder from the Transformers model, and is trained on 31 million protein sequences from Pfam53 [47]. The model’s effectiveness was validated across six downstream tasks including remote homology detection, contact prediction, and protein engineering tasks.

Considering that antibody is different from general proteins, for antibody embeddings, we used AbLang for embeddings [48]. AbLang is an antibody-specific language model trained on the Observed Antibody Space (OAS) database [49, 50], which contains about 71.98 million sequence data (52.89 million unpaired heavy chains and 19.09 million unpaired light chains). It can be used for antibody residue repair, sequence-specific predictions, and residue sequence embedding representations, and AbLang provides more accurate antibody representation than ESM-1b [51, 52]. Interestingly, AbLang requires separate embeddings for the heavy and light chains of the antibody, as two AbLang models were trained, one for the heavy chain and the other for the light chain. One potential reason for training separate models is that heavy and light chains have different components: the light chain has two such immunoglobulin domains, whereas the heavy chain of the antibody contains four.

### ConvNeXt backbone

The ConvNeXt network is composed purely of convolutional layers and inspired by the architecture of vision transformer and ResNet [53–55]. ConvNeXt mainly improves the model performance in the following aspects: (1) Macro design (2) ResNeXt (3) Reverse bottleneck (4) Large kernel size (5) Various layered micro designs. Overall, the ConvNeXt network has four stages, with a block stacking ratio of 1:1:3:1 for each stage, and a convolutional layer with the same kernel size of 4 and a step size as the Swin-T network. The ConvNeXt network has also designed an anti-bottleneck structure based on “fine end coarse medium” as a reference, replacing RELU with more commonly used GELU activation function, resulting in a slight improvement in model performance. ConvNeXt not only reduces the use of regularization functions, but also replaces Batch Norm with Layer Norm. These two operations slightly improve the accuracy of the model. In this study, we removed the final MLP layer of the original ConvNeXt backbone as one of the modules for DG-Affinity.

### Architecture of DG-Affinity

**Fig. 2:**
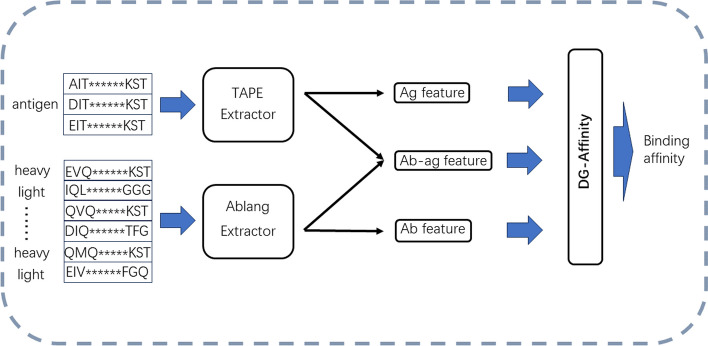
The workflow of DG-Affinity. Among them, Ab represents antibodies and Ag represents antigens. The antibody and antigen sequences are respectively fed into the corresponding embedding extractors, and three features are obtained: Ab features, Ag features, and Ab-ag features. Then, these three features are concatenated into the MLP to predict binding affinity values

**Fig. 3:**
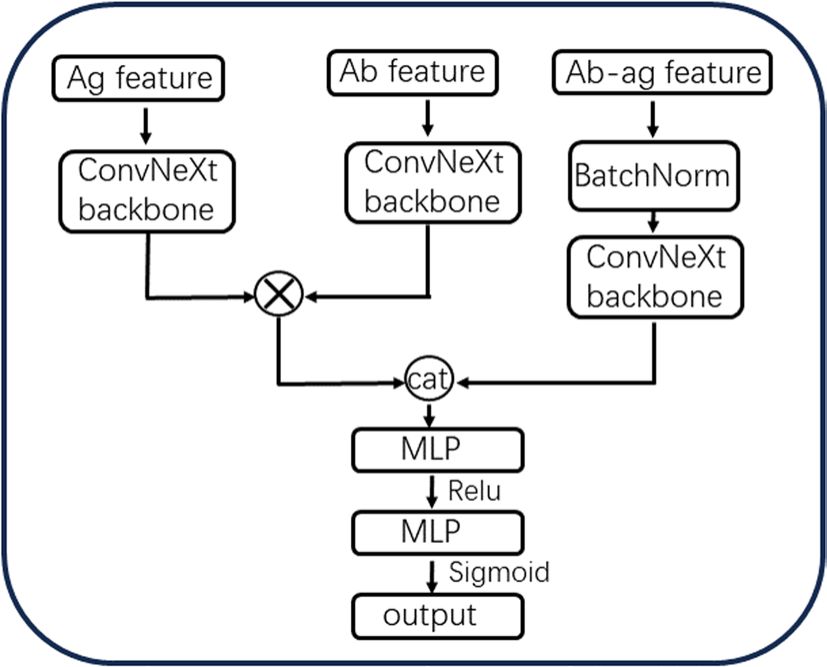
Network architecture of DG-Affinity, “cat” symbol represents concatenation, “$\times$” symbol represents element-wise multiplication, “Ag feature” is antigen feature, “Ab feature” is antibody feature, “Ab–ag feature” is concatenated feature of antigen feature and antibody feature and MLP is a single linear layer

Our DG-Affinity’s architecture is shown in Figs. 2 and 3, three parallel ConvNeXt backbones accept three different features obtained from TAPE or Ablang feature extractors, which are antibody features, antigen features, and antibody–antigen concatenated features. After passing through the ConvNeXt backbone for feature representation learning, the antibody representation and antigen representation are multiplied element-wise and concatenated with the antibody–antigen feature representation learned through the ConvNeXt backbone. Finally, the new representation is passed through two layers of MLP to predict the affinity value, among them, each layer of MLP is composed of a linear function followed by relu or sigmoid activate function. In this study, we used the open-source code to construct an ConvNeXt backbone (https://github.com/facebookresearch/ConvNeXt). Considering the capacity and performance of the model, we chose the tiny ConvNeXt version. The neural network was built and trained using the Pytorch library [56], and we trained the model using 50 epochs with a learning rate of 0.000001, using the ADAM optimizer [57]. The network output is a regression value prediction of the affinity.

### Performance metrics

Our model’s predictive ability was measured using the Pearson correlation coefficient (R), R-squared (R2), Root Mean Square Deviation (RMSD) and Mean Absolute Error (MAE). To better evaluate our model, we also tested other well-known structure-based antigen–antibody affinity prediction model (e.g. CSM-AB and AREA-AFFINITY). We input the 26 complex structures from the independent set into their online prediction website and manually calculated the R, R2, RMSD and MAE values based on the true and predicted binding affinity values for each method, respectively.

### Baseline methods

*CSM-AB* It is the first scoring function specifically designed for antibody antigen docking and binding affinity prediction. By adjusting the graph-based structure, this method can capture close-contact features and surrounding structural information related to antibody antigen binding.

*AREA-AFFINITY* It integrates 60 effective area-based protein–protein affinity prediction models and 37 effective area models for antibody protein antigen binding affinity prediction.

*LISA* It is an empirical affinity function based on the atomic-atomic contact model and a radial function based on the density functional theory.

*CIPS* It is a new pair potential combining interface composition with residue–residue contact preference.

*Prodigy* It is based on the counting of atom–atom contacts at the interface and on the charge distribution at the non-interacting surface of the complex.

*NIS* It considers distinguished physico-chemical properties of residues lying on the complex surface.

*CCharPPI* It integrates over a hundred tools into a single web server, including electrostatic, solvent removal, and hydrogen bonding models, as well as interface filling and complementarity scores, empirical potentials of various resolutions, docking potentials, and composite scoring functions.

We follow the comparison protocol of CSM-AB and LISA [58]. In the experiment, LISA, CIPS [59], NIS [60] and PRODIGY [61] were standalone scripts, AREA-AFFINITY [40] and CSM-AB were one-line webserver, other models or tools (e. g. FIREDOCK) were calculated using CCharPPI webserver based on physical potentials and composite descriptors [62]. We collected structure data from SAbDab database and Protein Data Bank since most of the existing comparative models are based on structure [63, 64].

## Results

### Comparison DG-Affinity with other baseline methods

**Fig. 4:**
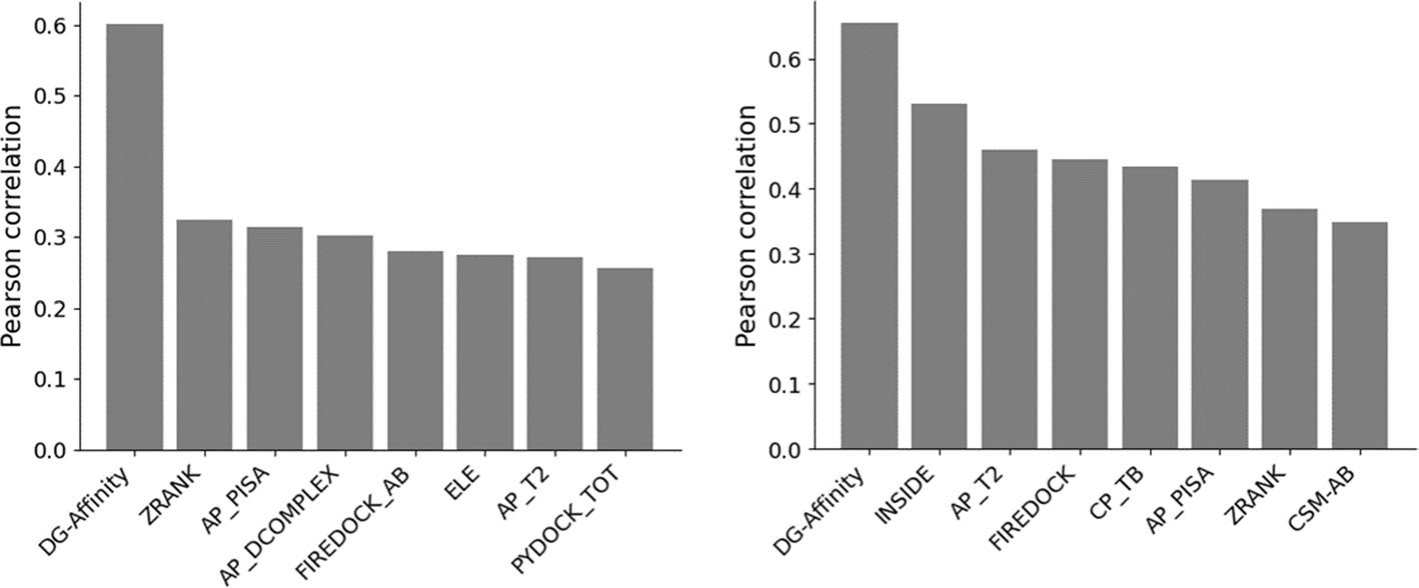
performance distribution of the top 8 models on the independent set (**a**) and the training set (**b**), respectively

To demonstrate the advantages of DG-Affinity, we evaluated other methods on the training set, the results in Fig. 4a and Additional file 1: Table S1 show that DG-Affinity significantly outperformed all of them. At the same time, we also evaluate the stability and consistency of our model’s performance using tenfold cross validation, 15-fold cross validation, and 20-fold cross validation. The results are stable and consistent (shown in Additional file 1: Figure S1), showing that there is no significant sampling bias for DG-Affinity. After manually adjusting the model parameters to the best performance on the validation set, we compared it with baseline methods on the independent test set. As shown in Fig. 4b and Additional file 1: Table S2, DG-Affinity achieves the best R of over 0.6556 in the independent set. Most of the baseline models yield an R from 0.3 to 0.5, which is much lower than that of DG-Affinity. Moreover, two models have negative Pearson’s correlation, such as CP_PIE (-0.3332) and AREA-AFFINITY (-0.2019). In addition, our method outperforms other methods in all the metrics (Additional file 1: Table S2).

#### Comparing the effectiveness of different architectures in DG-Affinity

**Fig. 5:**
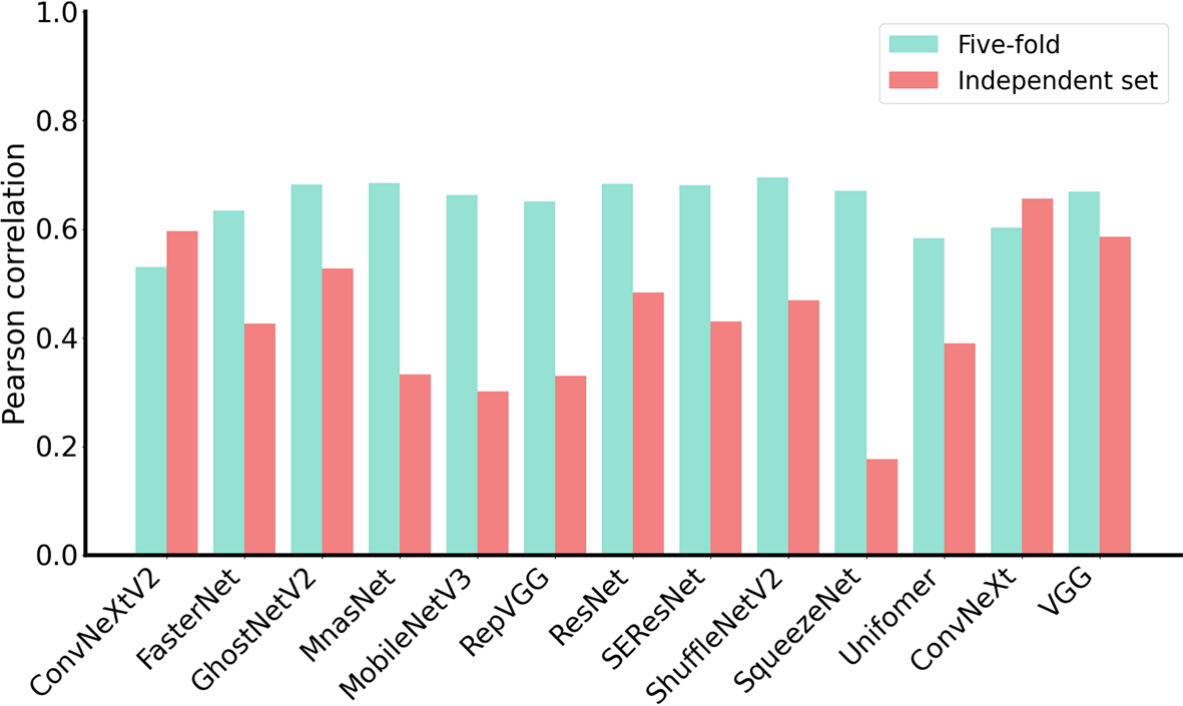
Performance of different network backbones in DG-Affinity on the training set (five-fold) and independent set

DG-Affinity uses ConvNeXt as the backbone network, to demonstrate the effectiveness, we compare it with the other 12 widely used backbone networks, i.e. convolutional network, transformer, and several classic models. The source codes for these architectures are downloaded from the corresponding github repository (https://github.com/weiaicunzai/pytorch-cifar100), and slightly modified to remove the final MLP layer and replace it with the global mean pooling. These backbones replace all ConvNeXt modules in DG-Affinity, and the replaced model structure is trained and validated using the aforementioned training set and independent test set. For detailed hyperparameter information, please refer to Additional file 1: Table S3 in the supporting material. The results are shown in Fig. 5 and Additional file 1: Table S4, S5. Of the 13 network backbones, the ConvNeXt achieves relatively better performance than other backbone networks in the fivefold cross validation and in the independent test dataset.

#### Exploration of model ablation studies

**Fig. 6:**
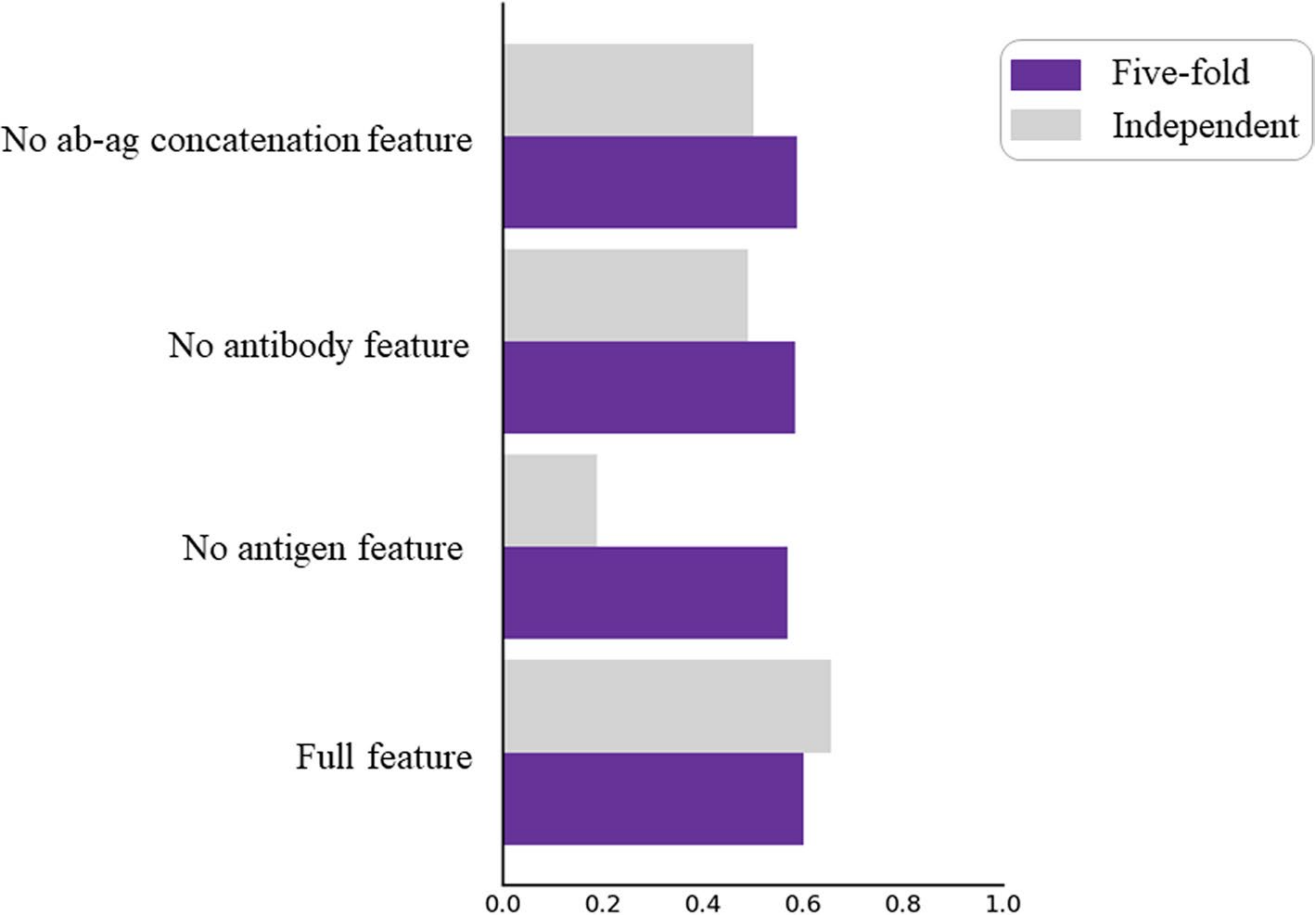
The ablation experiments of our model on the training set (five-fold) and independent set. The results of model in training and independent sets are represented by purple and gray bars

In this section, we investigated the contribution of each module in DG-Affinity. During the experiment, we maintain the parameter consistency. After respectively removing the ConvNeXt module for learning antigen features, antibody features, and concatenated features antibody and antigen embeddings, we concatenate the output representation vectors of these remaining ConvNeXt modules and input them into the MLP layer to make regression. As shown in the Fig. 6, it is evident that the lack of the module for learning antigen features has a significant impact on the performance of DG-Affinity. The results show that antigen information may be more closely related to the interactions than antibodies. We have also found that when using full feature, the performance on the independent datasets in Fig. 5 is higher than that of five-fold. One potential reason is that the independent datasets are not large enough and the samples are imbalanced, a more number of features potentially introduce better generalizability, resulting in a better performance on the independent test set.

## Discussion

The development of bioengineering has led the development of antibody drugs, demonstrating good therapeutic effects in the treatment of cancer and autoimmune diseases. The key step in this development process is to obtain the affinity between antibodies and their binding antigens. However, the limitation of current methods lies in the requirement for structural information of antigen antibody complexes, which is difficult to obtain. To address the above issues, in this study, we propose a deep learning based model for predicting antigen–antibody binding affinity with pre-trained emebddings from sequences, our model has achieved promising performance due to the followings: (1) the training dataset is larger, while traditional structure-based methods have much smaller structure data set than sequences, making them prone to overfitting and low generalization performance. (2) The ConvNeXt framework has recently been widely used in various fields and has been proven to achieve good prediction results. (3) The pre-trained emeddings on the large unlabeled sequences for proteins and antibody.

## Conclusion

In this study, we propsoed a new sequence-based antigen–antibody binding affinity prediction method, named DG-Affinity, based on protein and antibody language models. Antigen and antibody sequences are first transformed into the embedding vectors through two pre-trained methods (TAPE and Ablang), then a ConvNeXt-backbone based network is used to learn the affinity relationship between antigen and antibody. The results on benchmark datasets indicate that DG-Affinity outperforms existing methods, including the popular structure-based antigen antibody affinity prediction methods as well as the traditional tools, for both the fivefold and independent validation, achieving a Pearson correlation coefficient of over 0.65 on the independent test datasets. In addition, we developed an easy-to-use website version of DG-Affinity. It can be expected that our method DG-Affinity will advance the progress and development of antibody drug.

### Supplementary Information

**Additional file 1: Table S1.** Performance comparation between DG-Affinity and other models on training set. **Table S2.** Performance comparation between DG-Affinity and other models on independent set. **Table S3.** Parameter settings for each model backbone on DG Affinity. **Table S4.** Performance of DG-Affinity with different backbones on 5-fold cross validation. **Table S5.** Performance of DG-Affinity with different backbones on independent datasets. **Fig. S1.** Performance distribution of DG-Affinity per fold under different validation schemes including, 5-fold, 10-fold and 20-fold cross validation, Prove the consistency and robustness of this method.

## Data Availability

The website is provided at https://www.digitalgeneai.tech/solution/affinity, this study does not include any human involvement or human data which is not publicly available.

## Abbreviations

CDRs: Complementarity-determining regions
MAbs: Monoclonal antibodies
ADCs: Antibody–drug conjugates
RIA: Adioimmunoassay
ELISA: Enzyme-linked immunosorbent assay
SPR: Surface Plasmon resonance
BLI: Bio-layer interferometry
GRUs: Bidirectional gated recursive unit module
PIPR: Protein–Protein Interaction Prediction Based on Siamese Residual RCNN
OAS: Observed Antibody Space
ESM: Evolutionary Scale Modeling
