# Supplementary material for: DG-Affinity: predicting antigen–antibody affinity with language models from sequences
Source: BMC Bioinformatics. 2023 Nov 13;24:430. doi: 10.1186/s12859-023-05562-z (PMC10644518; doi:10.1186/s12859-023-05562-z)
Supplement: Supplementary file 1 — Additional file 1: Table S1. Performance comparation between DG-Affinity and other models on training set. Table S2. Performance comparation between DG-Affinity and other models on independent set. Table S3. Parameter settings for each model backbone on DG Affinity. Table S4. Performance of DG-Affinity with different backbones on 5-fold cross validation. Table S5. Performance of DG-Affinity with different backbones on independent datasets. Fig. S1. Performance distribution of DG-Affinity per fold under different validation schemes including, 5-fold, 10-fold and 20-fold cross validation, Prove the consistency and robustness of this method. [file 12859_2023_5562_MOESM1_ESM.docx]

**SUPPLEMENTARY MATERIALS**

DG-Affinity: Predicting Antigen-Antibody Affinity with language models from Sequences

Table S1. Performance comparation between DG-Affinity and other models on training set

| **Methods** | **R** | **R2** | **RMSD** | **MAE** |
| --- | --- | --- | --- | --- |
| CIPS-freesasa | -0.0812 | -35.5196 | 11.9712 | 11.8056 |
| CIPS-naccess | -0.0805 | -35.5179 | 11.9710 | 11.8053 |
| NIS | -0.0687 | -193.0096 | 27.5923 | 27.3643 |
| PRODIGY | 0.1478 | -0.7136 | 2.5932 | 2.0338 |
| LISA | 0.2328 | -0.1287 | 2.1046 | 1.6622 |
| AP_DCOMPLEX | 0.3023 | -5.1368 | 4.9073 | 3.8186 |
| AP_DFIRE2 | 0.0592 | -170275.3160 | 817.4374 | 131.4154 |
| AP_PISA | 0.3145 | -30.4824 | 11.1150 | 10.9444 |
| AP_T2 | 0.2716 | -672.8094 | 51.4216 | 46.0786 |
| AP_dDFIRE | 0.0580 | -230443.1373 | 950.9556 | 147.7216 |
| CP_DDG_W | 0.1798 | -8.5400 | 6.1186 | 4.8251 |
| CP_TB | 0.2447 | -295.6337 | 34.1183 | 29.7565 |
| ELE | 0.2753 | -33.3912 | 11.6172 | 8.3770 |
| FIREDOCK | 0.2159 | -96.7166 | 19.5822 | 15.9130 |
| FIREDOCK_AB | 0.2802 | -856.9465 | 58.0239 | 53.1524 |
| HBOND2 | 0.1520 | -10.5573 | 6.7345 | 6.0176 |
| LK_SOLV | -0.1977 | -662.1969 | 51.0150 | 48.6608 |
| PYDOCK_TOT | 0.2559 | -160.5253 | 25.1766 | 21.3334 |
| ROSETTADOCK | 0.1363 | -17.5435 | 8.5304 | 4.7822 |
| ZRANK | 0.3252 | -1663.9615 | 80.8312 | 75.1357 |
| ZRANK2 | 0.1764 | -20924.7751 | 286.5617 | 253.2272 |
| INSIDE | -0.1733 | -194.8153 | 27.7204 | 26.9836 |
| CP_PIE | -0.2863 | -40.6138 | 12.7789 | 12.5948 |
| SIPPER | -0.1544 | -93.7545 | 19.2831 | 17.9309 |
| **DG-Affinity** | **0.6023** | 0.3531 | 0.1329 | 0.0995 |

Table S2. Performance comparation between DG-Affinity and other models on independent set

| **Methods** | **R** | **R2** | **RMSD** | **MAE** |
| --- | --- | --- | --- | --- |
| AREA-AFFINITY | -0.2019 | -6.8468 | 5.2025 | 4.4687 |
| CSM-AB | 0.3210* | -1.9305 | 3.2808 | 2.3838 |
| CIPS-freesasa | 0.3035 | -38.4553 | 11.6659 | 11.5189` |
| CIPS-naccess | 0.3034 | -38.4553 | 11.6659 | 11.5189 |
| NIS | 0.0047 | -232.3863 | 28.3729 | 28.2467 |
| PRODIGY | 0.2414 | -1.1889 | 2.7477 | 2.2626 |
| LISA | 0.3451 | -0.1409 | 1.9838 | 1.6302 |
| AP_DCOMPLEX | 0.1718 | -4.8428 | 4.4893 | 3.4817 |
| AP_DFIRE2 | -0.0039 | -124.4625 | 20.8028 | 17.7696 |
| AP_PISA | 0.4136 | -39.6771 | 11.8451 | 11.7077 |
| AP_T2 | 0.4592 | -678.8682 | 48.4260 | 44.2130 |
| AP_dDFIRE | -0.0176 | -134.1086 | 21.5877 | 13.4880 |
| CP_DDG_W | 0.2933 | -4.7939 | 4.4704 | 3.3652 |
| CP_TB | 0.4343 | -314.2330 | 32.9748 | 31.1253 |
| ELE | 0.2848 | -82.8697 | 17.0086 | 12.9177 |
| FIREDOCK | 0.4443 | -113.0402 | 19.8333 | 15.4311 |
| FIREDOCK_AB | 0.3451 | -1059.7732 | 60.4891 | 53.9423 |
| HBOND2 | 0.1946 | -12.6225 | 6.8548 | 5.9409 |
| LK_SOLV | -0.2527 | -954.9959 | 57.4241 | 55.8438 |
| PYDOCK_TOT | 0.3048 | -167.7346 | 24.1250 | 20.6391 |
| ROSETTADOCK | 0.1773 | -9.5973 | 6.0459 | 5.3280 |
| ZRANK | 0.3682 | -2257.3393 | 88.2594 | 82.3089 |
| ZRANK2 | 0.3442 | -22139.9066 | 276.3529 | 254.9132 |
| INSIDE | 0.5301 | -270.7322 | 30.6151 | 30.4846 |
| CP_PIE | -0.3332 | -52.5285 | 13.5881 | 13.4345 |
| SIPPER | -0.3330 | -94.4527 | 18.1451 | 16.9930 |
| **DG-Affinity** | **0.6556** | 0.3670 | 0.1172 | 0.0962 |

***PS: Due to Internal Server Error, CSM-AB was unable to obtain results 5 out of 26 complexes.***

Table S3. Parameter settings for each model backbone on DG Affinity

| **Backbones** | **Version** | **Batch size** | **Dimension*** |
| --- | --- | --- | --- |
| ConvNeXtV2 [1] | tiny | 8 | 1536 |
| FasterNet [2] | default | 8 | 1024 |
| GhostNetV2 [3] | default | 8 | 2560 |
| MnasNet [4] | default | 8 | 2560 |
| MobileNetV3 [5] | small | 8 | 1152 |
| RepVGG [6] | a0 | 8 | 2560 |
| ResNet | resnet-16 | 8 | 1024 |
| SEResNet [7] | seresnet-18 | 4 | 1024 |
| ShuffleNetV2 [8] | default | 8 | 2048 |
| SqueezeNet [9] | default | 8 | 1024 |
| Unifomer [10] | small | 8 | 1024 |
| ConvNeXt | tiny | 8 | 1536 |
| VGG [11] | vgg -16 | 8 | 1024 |

“*****” refer to input dimension of the second to last MLP

Table S4. Performance of DG-Affinity with different backbones on 5-fold cross validation.

| **Backbones** | **R** | **R2** | **MAE** | **RMSE** |
| --- | --- | --- | --- | --- |
| ConvNeXtV2 | 0.5297 | 0.2524 | 0.1088 | 0.1430 |
| FasterNet | 0.6334 | 0.3235 | 0.1051 | 0.1358 |
| GhostNetV2 | 0.6815 | 0.4206 | 0.0915 | 0.1259 |
| MnasNet | 0.6843 | 0.4505 | 0.0893 | 0.1225 |
| MobileNetV3 | 0.6618 | 0.4138 | 0.0910 | 0.1266 |
| RepVGG | 0.6505 | 0.4111 | 0.0913 | 0.1269 |
| ResNet | 0.6823 | 0.4407 | 0.0912 | 0.1235 |
| SEResNet | 0.6796 | 0.4423 | 0.0897 | 0.1234 |
| ShuffleNetV2 | **0.6950** | 0.4680 | 0.0879 | 0.1204 |
| SqueezeNet | 0.6694 | 0.4145 | 0.0917 | 0.1263 |
| Unifomer | 0.5824 | 0.3148 | 0.1024 | 0.1368 |
| ConvNeXt | 0.6023 | 0.3531 | 0.0995 | 0.1329 |
| VGG | 0.6680 | 0.4290 | 0.0919 | 0.1248 |

Table S5. Performance of DG-Affinity with different backbones on independent datasets.

| **Backbones** | **R** | **R2** | **MAE** | **RMSE** |
| --- | --- | --- | --- | --- |
| ConvNeXtV2 | 0.5957 | 0.1340 | 0.1105 | 0.1367 |
| FasterNet | 0.4257 | 0.0203 | 0.1154 | 0.1457 |
| GhostNetV2 | 0.5272 | 0.1794 | 0.1087 | 0.1325 |
| MnasNet | 0.3327 | -0.0262 | 0.1199 | 0.1488 |
| MobileNetV3 | 0.3009 | 0.0061 | 0.1177 | 0.1470 |
| RepVGG | 0.3299 | 0.0385 | 0.1215 | 0.1441 |
| ResNet | 0.4828 | 0.1586 | 0.1110 | 0.1341 |
| SEResNet | 0.4292 | 0.1492 | 0.1102 | 0.1353 |
| ShuffleNetV2 | 0.4689 | 0.1898 | 0.1043 | 0.1326 |
| SqueezeNet | 0.1768 | -0.0314 | 0.1159 | 0.1497 |
| Unifomer | 0.3888 | 0.0271 | 0.1181 | 0.1451 |
| ConvNeXt | **0.6557** | 0.3670 | 0.0962 | 0.1172 |
| VGG | 0.5860 | 0.3104 | 0.0974 | 0.1221 |


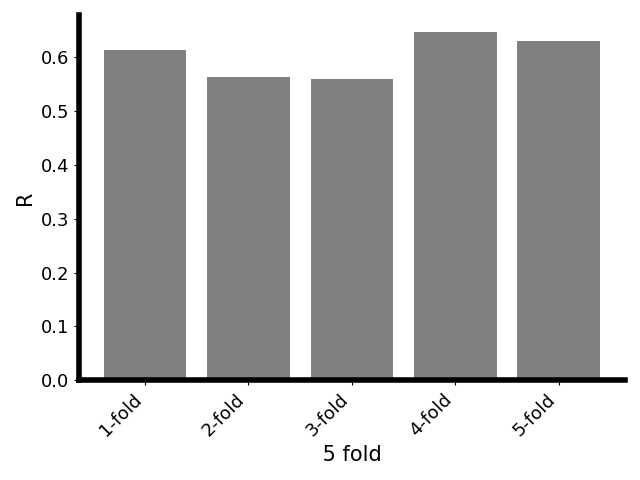

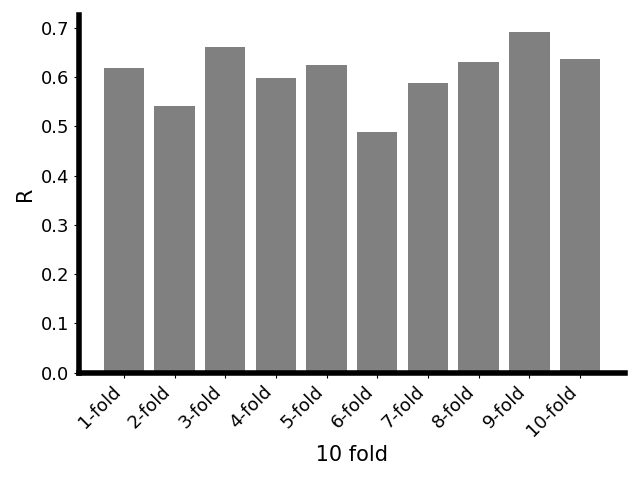


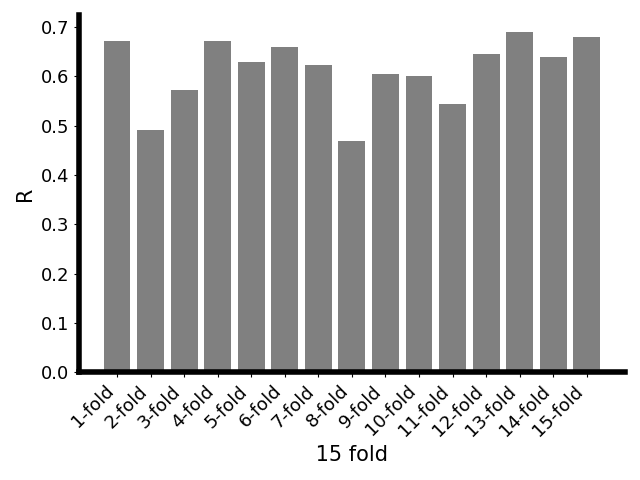

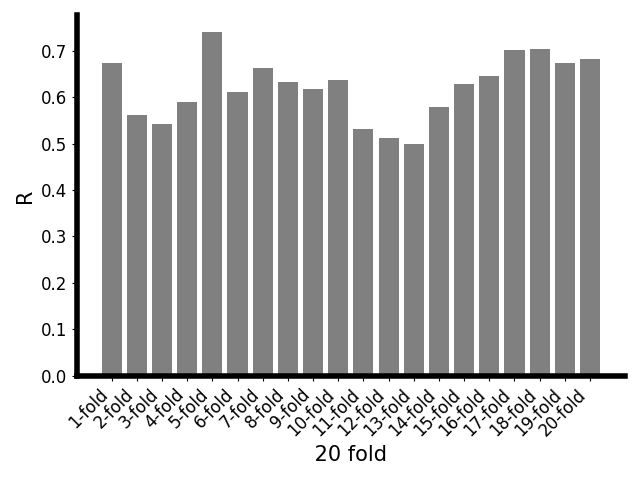


**Fig.S1** Performance distribution of DG-Affinity per fold under different validation schemes including, 5-fold, 10-fold and 20-fold cross validation, Prove the consistency and robustness of this method.

**References**

1. Woo S, Debnath S, Hu R, et al. Convnext v2: Co-designing and scaling convnets with masked autoencoders. Proceedings of the IEEE/CVF Conference on Computer Vision and Pattern Recognition. 2023: 16133-16142.

2. Chen J, Kao S, He H, et al. Run, Don't Walk: Chasing Higher FLOPS for Faster Neural Networks. Proceedings of the IEEE/CVF Conference on Computer Vision and Pattern Recognition. 2023: 12021-12031.

3. Han K, Wang Y, Xu C, et al. GhostNets on heterogeneous devices via cheap operations. International Journal of Computer Vision, 2022, 130(4): 1050-1069.

4. Tan M, Chen B, Pang R, et al. Mnasnet: Platform-aware neural architecture search for mobile. Proceedings of the IEEE/CVF conference on computer vision and pattern recognition. 2019: 2820-2828.

5. Howard A, Sandler M, Chu G, et al. Searching for mobilenetv3[C]//Proceedings of the IEEE/CVF international conference on computer vision. 2019: 1314-1324.

6. Ding X, Zhang X, Ma N, et al. Repvgg: Making vgg-style convnets great again[C]//Proceedings of the IEEE/CVF conference on computer vision and pattern recognition. 2021: 13733-13742.

7. Hu J, Shen L, Sun G. Squeeze-and-excitation networks. Proceedings of the IEEE conference on computer vision and pattern recognition. 2018: 7132-7141.

8. Ma N, Zhang X, Zheng H T, et al. Shufflenet v2: Practical guidelines for efficient cnn architecture design. Proceedings of the European conference on computer vision (ECCV). 2018: 116-131.

9. Iandola F N, Han S, Moskewicz M W, et al. SqueezeNet: AlexNet-level accuracy with 50x fewer parameters and< 0.5 MB model size. arXiv preprint arXiv:1602.07360, 2016.

10. Li K, Wang Y, Zhang J, et al. Uniformer: Unifying convolution and self-attention for visual r ecognition[J]. IEEE Transactions on Pattern Analysis and Machine Intelligence, 2023.

11. Simonyan K, Zisserman A. Very deep convolutional networks for large-scale image recognition[J]. arXiv preprint arXiv:1409.1556, 2014.
